# Genetic alterations in the classification of tumors of the haematopoietic system: From guiding diagnosis to instructing treatment in leukaemias and lymphomas

**DOI:** 10.1515/medgen-2024-2002

**Published:** 2024-03-06

**Authors:** German Ott, Claudia Haferlach, Reiner Siebert

**Affiliations:** MLL – Münchner Leukämielabor München Germany; Robert-Bosch-Krankenhaus Abteilung für Klinische Pathologie Stuttgart Germany; Universität Ulm & Universitätsklinikum Ulm Institut für Humangenetik Ulm Germany

With the emergence of the 5^th^ edition of the World Health Organization (WHO) Classification of Haematolymphoid Tumours (WHO-HAEM5) [1,2], we again witness a groundbreaking change in the characterization of haematolymphoid tumours, facing an ever-growing impact of genetic information in the classification, their importance in the risk stratification and ultimately therapy of these tumours. In this special issue of the *Medizinische Genetik*, we provide a comprehensive overview of genetic alterations important in the classification of haematopoietic neoplasms in WHO-HAEM5 and their relevance in myeloid and lymphoid neoplasms. This issue highlights key topics that encompass the appraisal of current technologies, practical guides to genetic studies, and insights into germline predispositions in haematologic malignancies.

In the initial chapter *Overview on WHO-HAEM5 and the diagnostic relevance of genetic alterations for the classification* we provide an introduction to the role genetic diagnostics has gained since the last revision of the WHO classification back in 2017. In the subsequent second introductory article, *Appraisal of current technologies for genetic exploration,* Salaverria and colleagues scrutinize the latest technical advancements in genetic analysis that empower us to comprehensively profile the genetic landscape of haematological malignancies. Hörst and associates explore the role of genetics in the continuum of *Clonal haematopoiesis, myelodysplastic neoplasms (MDS) and acute myeloid leukemia*. Kühn and colleagues describe the nature and impact of the genetic landscape in the heterogeneous families of *myeloproliferative neoplasms (MPN) and myelodysplastic/myeloproliferative neoplasms* (MDS/MPN). After these thorough overviews on the importance of genetic markers for the diagnosis of tumours of the myeloid lineage, the role of genetic alterations in the classification and risk stratification of *Lymphoblastic Leukaemias/Lymphomas (ALL/LBL)* is described by Steinemann et al. Switching from precursor to mature lymphoid malignancies, Tausch and colleagues examine the nature and role of genetic alterations in *Chronic Lymphocytic Leukaemia* including its pre-neoplastic lesion, *Monoclonal B-Cell Lymphocytosis (MBL) and Plasma Cell Neoplasms*. Finally, the genetic landscape of the heterogeneous group of *mature B- and T-Cell Lymphomas* is presented by López and associates who detail the importance of genetic alterations both in B-cell and T-cell lymphoid neoplasms.

This special issue, therefore, provides a unique compilation of new and exciting data centered on new concepts of the WHO-HAEM5. At the same time, application of these genetic data validates the concepts that were laid down in this new – 5^th^ – volume of the Classification of Haematolymphoid Neoplasms.

As can be seen from the different chapters of this special issue, the comprehensive and up-to-date diagnosis of tumors of the haematolymphatic system is a true interdisciplinary challenge. Therefore, as is the case for WHO-HAEM5, also this special issue has been compiled by a multidisciplinary author team which includes haematopathologists, haemato-oncologists and scientists – besides geneticists. We are grateful to several editors and authors of WHO-HAEM5, who – along with other leading colleagues in the field – contributed to this issue as expert authors or as reviewers providing helpful input.

“*We are grateful to – and stand on the shoulders of – countless individuals and research teams, who have contributed significantly to establish the foundations of the current lymphoma classification*.” … states the epilogue of one of the papers with the initial description of WHO-HAEM5 [1]. We would like to extend this gratitude to the many renowned (and less well-known) colleagues associated with the German Society of Human Genetics who were former frontrunners in the field of leukaemia and lymphoma genetics, with Lore Zech, Christa Fonatsch, Brigitte Schlegelberger, Jochen Harbott and Oskar Haas being named here representative of many others.
